# The Nuremberg Code–A critique

**DOI:** 10.4103/2229-3485.80371

**Published:** 2011

**Authors:** Ravindra B. Ghooi

**Affiliations:** *Bilcare Research Academy, Sai Capital, Senapati Bapat Marg, Pune, Maharashtra, India*

**Keywords:** Ethics, guidelines, human subjects, Nuremberg, research

## Abstract

The Nuremberg Code drafted at the end of the Doctor’s trial in Nuremberg 1947 has been hailed as a landmark document in medical and research ethics. Close examination of this code reveals that it was based on the Guidelines for Human Experimentation of 1931. The resemblance between these documents is uncanny. It is unfortunate that the authors of the Nuremberg Code passed it off as their original work. There is evidence that the defendants at the trial did request that their actions be judged on the basis of the 1931 Guidelines, in force in Germany. The prosecutors, however, ignored the request and tried the defendants for crimes against humanity, and the judges included the Nuremberg Code as a part of the judgment. Six of ten principles in Nuremberg Code are derived from the 1931 Guidelines, and two of four newly inserted principles are open to misinterpretation. There is little doubt that the Code was prepared after studying the Guidelines, but no reference was made to the Guidelines, for reasons that are not known. Using the Guidelines as a base document without giving due credit is plagiarism; as per our understanding of ethics today, this would be considered unethical. The Nuremberg Code has fallen by the wayside; since unlike the Declaration of Helsinki, it is not regularly reviewed and updated. The regular updating of some ethics codes is evidence of the evolving nature of human ethics.

## INTRODUCTION

The Nuremberg Code has served as a foundation for ethical clinical research since its publication 60 years ago. This landmark document, developed in response to the horrors of human experimentation done by Nazi physicians and investigators, focused crucial attention on the fundamental rights of research participants and on the responsibilities of investigators. It was prepared at a very momentous occasion, following the formal surrender of Germany at the end of the Second World War.

The Allied Commanders were well aware of the atrocities committed by the German Forces, on civilians and prisoners of war, and prosecuted the leading German authorities. Popularly known as the Nuremberg Trials, these were a series of military tribunals, held by the victorious Allied forces, wherein prominent members of the political, military, and economic leadership of the defeated Nazi Germany were prosecuted. The trials were held in the city of Nuremberg, Bavaria, Germany, in 1945-1946, at the Palace of Justice.

The first and best known of these trials was the Trial of the Major War Criminals before the International Military Tribunal, which tried 22 of the most important captured leaders of Nazi Germany, though several key architects of the war (such as Adolf Hitler, Heinrich Himmler and Josef Goebbels) had committed suicide before the trials began. The initial trials were held between November 20, 1945 and October 1, 1946. These trials are graphically described by Albert Speer in his book “Spandau-The Secret Diaries.”[1]

## DOCTOR’S TRIAL IN NUREMBERG

The second set of trials of lesser war criminals was conducted under Control Council Law No. 10 at the US Nuremberg Military Tribunals; among them was included the Doctors’ Trial which is most relevant to the Nuremberg Code. The judges in this case were Walter B. Beals (presiding judge) from Washington, Harold L. Sebring from Florida, and Johnson T. Crawford from Oklahoma, with Victor C. Swearingen, a former special assistant to the Attorney General of the United States, as an alternate judge. The Chief of Counsel for the Prosecution was Brig Gen Telford Taylor and the chief prosecutor James M. McHaney. The indictment was filed on October 25, 1946; the trial lasted from December 9 that year until August 20, 1947. Of the 23 defendants, 20 were doctors, while 3 were administrators.

The accused were indicted on four counts viz.

- Count I--The Common Design or Conspiracy
- Count II--War Crimes
- Count III--Crimes against Humanity
- Count IV--Membership in a Criminal Organization

Interestingly, there was one woman among them and she was Herta Oberheuser, who was convicted to 20 years in prison (later reduced to 10 years on appeal), on count II and III.

In his opening statement for prosecution,[2] Brig Gen Telford Taylor described the medical set up in Germany and the affiliation of defendants to the different arms and services. He then went on to relate the counts on which each of the defendants was indicted and finally spoke of violation of Medical ethics. Taylor stated that the 20 physician defendants had violated the Hippocratic Oath including its fundamental principle “Primum non nochere.” Taylor said that on November 24, 1933, the Nazis passed a law to protect animals from being cruelly treated, and that animals should be used judiciously, only when necessary and finally put to death painlessly after the completion of the experiment. He alleged that defendants behaved with less humanity toward fellow humans than was demanded by the animal protection law.

The charge against the 20 defendants was that they had violated the Hippocratic Oath and behaved in a manner incompatible with their education and profession. The defendants were charged with war crimes and crimes against humanity. The prosecution’s case rested upon what was considered ethical in general by the medical profession and the world, though no specific code for medical research existed (as per the prosecutors).

What is strange is that nowhere in his opening statement did Brig General Taylor or the Chief Prosecuting attorney (James M. McHaney) refer to the 1931 Guidelines for Human Experimentation passed by German Government. These guidelines for therapeutic and scientific research on human subjects were published originally as a Circular of the Reich Minister of the Interior dated February 28, 1931. The guidelines remained in force until 1948, but for unknown reasons, they were not included in the Omnibus Law (Ueberleitungsgesetz) passed by the Bundestag after 1948, which transported hundreds of laws and regulations of the Reich into the Federal Republic’s legal structure.

## THE 1931 GUIDELINES

The 1931 guidelines are claimed to be the first of their kind,[3] though there existed an older and briefer code called the Berlin Code 1900. The Berlin code was enacted by the Prussian Government, but subsequently, the Prussian Empire gave way to German Republic. The guidelines of 1931 included a number of points, the most important of which are as follows:

5. Innovative therapy may be carried out only after the subject or his legal representative has unambiguously consented to the procedure in the light of relevant information provided in advance. Where consent is refused, innovative therapy may be initiated only if it constitutes an urgent procedure to preserve life or prevent serious damage to health and prior consent could not be obtained under the circumstances.

6. The question of whether to use innovative therapy must be examined with particular care where the subject is a child or a person under 18 years of age.

10. A report shall be made in respect of any innovative therapy, indicating the purpose of the procedure, the justification for it, and the manner in which it is carried out. In particular, the report shall include a statement that the subject or, where appropriate, his legal representative has been provided in advance with relevant information and has given his consent.

Where therapy has been carried out without consent, under the conditions referred to in the second Paragraph of Section 5, the statement shall give full details of these conditions.

These guidelines were issued by Weimar Government which replaced the Imperial Government in 1919, however, the government that issued the guidelines did not survive long. The Nazi party under Adolf Hitler took over the reigns of the government on January 30, 1933, and this government ignored the guidelines. Historically, the guidelines were never repealed and they remained on statute books till 1948. The Guidelines were both well written and elaborated on the Berlin Code of 1900 that had been passed by the Prussian Government.[4]

The defendants in the 1946 trial could have been tried for violation of the Guidelines for Human Experimentation, but they were not. The prosecutors appeared to be ignorant of these Guidelines, though it is claimed that the defendants requested that they be tried under these guidelines.[5]

The Nuremberg Code was a hastily put together document on the advice of medical experts who took part in the trial. It is believed that Harold Sebring was the author of the Code. The two American physicians who helped prosecute the Nazi doctors at Nuremberg, Leo Alexander, and Andrew Ivy, have also been identified as the Code’s authors. A careful reading of the transcript of the Doctors’ Trial, background documents, and the final judgment reveals that authorship was shared and that the famous 10 principles of the Code grew out of the trial itself.[6]

Andrew Ivy, the American physiologist who assisted the court at the Doctor’s trial and suggested at least three points for the Nuremberg Code, claimed during cross examination that there were no written principles of research in the United States or elsewhere before December 1946. This statement, at best, was misrepresentation of facts and at worst an act of perjury.

It is acknowledged world wide that the Code does not have the force of law behind it. Each principle enunciated in this code has been widely acclaimed and explained but the deficiencies in the code have not been commented upon. There are some glaring errors, some wrong usage of language which need to be understood, in order to appreciate why this document, so revered and respected, fails to fulfill any purpose at all. Additionally, the Nuremberg Code is a document which has copied ideas from the 1931 guidelines, and since it has been done without acknowledging the source, is guilty of plagiarism.

## COMPARISON OF THE CODES

A point-by-point comparison of the code with the guidelines shows that the code was based on 1931 guidelines, and often the guidelines were not interpreted correctly [Table 1]

**Table 1:** Comparison of the Nuremberg Code with the 1931 Guidelines

The Nuremberg Code	1931 Guidelines

1. The voluntary consent of the human subject is absolutely essential. This means that the person involved should have legal capacity to give consent; should be situated as to be able to exercise free power of choice, without the intervention of any element of force, fraud, deceit, duress, over-reaching, or other ulterior form of constraint or coercion, and should have sufficient knowledge and comprehension of the elements of the subject matter involved as to enable him to make an understanding and enlightened decision. This latter element requires that before the acceptance of an affirmative decision by the experimental subject, there should be made known to him the nature, duration, and purpose of the experiment; the method and means by which it is to be conducted; all inconveniences and hazards reasonably to be expected; and the effects upon his health or person which may possibly come from his participation in the experiment. The duty and responsibility for ascertaining the quality of the consent rests upon each individual who initiates, directs, or engages in the experiment. It is a personal duty and responsibility which may not be delegated to another with impunity.	5. Innovative therapy may be carried out only after the subject or his legal representative has unambiguously consented to the procedure in the light of relevant information provided in advance. Where consent is refused, innovative therapy may be initiated only if it constitutes an urgent rocedure to preserve life or prevent serious damage to health and prior consent could not be obtained under the circumstances.
	7. Exploitation of social hardship in order to undertake innovative therapy is incompatible with the principles of medical ethics.

2. The experiment should be such as to yield fruitful results for the good of society, unprocurable by other methods or means of study, and not random and unnecessary in nature.	4. Any innovative therapy must be justified and performed in accordance with the principles of medical ethics and the rules of medical practice and theory. In all cases, the question of whether any adverse effects which may occur are proportionate to the anticipated benefits shall be examined and assessed. Innovative therapy may be carried out only if it has been tested in advance in animal trials (where these are possible).
3. The experiment should be so designed and based on the results of animal experimentation and knowledge of the natural history of the disease or other problem under study that the anticipated results will justify the performance of the experiment.	(b) Experimentation involving human subjects shall be avoided if it can be replaced by animal studies. Experimentation involving human subjects may be carried out only after all data that can be collected by means of those biological methods (laboratory testing and animal studies) that are available to medical science for purposes of clarification and confirmation of the validity of the experiment have been obtained. Under these circumstances, motiveless and unplanned experimentation involving human subjects shall obviously be prohibited.

4. The experiment should be so conducted as to avoid all unnecessary physical and mental suffering and injury.	Not covered by the 1931 Guidelines
5. No experiment should be conducted where there is an a priori reason to believe that death or disabling injury will occur; except, perhaps, in those experiments where the experimental physicians also serve as subjects.	

6. The degree of risk to be taken should never exceed that determined by the humanitarian importance of the problem to be solved by the experiment.	Risk benefit analysis covered under Point 4 of the guidelines

7. Proper preparations should be made and adequate facilities provided to protect the experimental subject against even remote possibilities of injury disability or death.	Not covered by the 1931 Guidelines

8. The experiment should be conducted only by scientifically qualified persons. The highest degree of skill and care should be required through all stages of the experiment of those who conduct or engage in the experiment.	9. In clinics, policlinics, hospitals, or other treatment and care establishments, innovative therapy may be carried out only by the physician in charge or by another physician acting in accordance with his express instructions and subject to his complete responsibility.

9. During the course of the experiment, the human subject should be at liberty to bring the experiment to an end if he has reached the physical or mental state where continuation of the experiment seems to him to be impossible.	Not covered by the 1931 Guidelines
10. During the course of the experiment, the scientist in charge must be prepared to terminate the experiment at any stage, if he has probable cause to believe, in the exercise of the good faith, superior skill, and careful judgment required by him that a continuation of the experiment is likely to result in injury, disability, or death to the experimental subject.	Not covered by the 1931 Guidelines


The 1931 Guidelines cover the Principles No. 4, 5, 9, and 10, but not in so many words. If the guideline is to be followed in spirit and not in word, a new code would be redundant.

## LOOPHOLES IN THE CODE

It should also be recalled that the 1931 guidelines predate the Code by a good 16 years, and these were very momentous years for the world. The medical world changed beyond recognition during this period, a reflection of which is seen in the code. So, also, the 1931 guidelines were in German, and translation may have taken away some of the bite that they had.

None the less, it is interesting to read the principles introduced in the Nuremberg Code, over and above what existed in the 1931 guidelines and analyze them.

Article 4. This is absolutely essential and there is no ambiguity in the language used.

Article 5. This article seems to suggest that studies that are endangering the life of subjects are permissible, if the investigator also takes is a subject. This runs against natural justice, just because the investigator is ready to risk his own life, he has no right to endanger another person’s life. By this token, a drunken pilot should be allowed to fly, since his own life is at jeopardy along with that of his passengers.

Article 9. This article is absolutely essential and noncontroversial.

Article 10. In principle, this article is essential, but its language is faulty. The investigator is not required to terminate the trial, but should be merely prepared to do so, if he/she thinks there is risk of death or serious injury to the subject. The difference between being required to stop and ready to stop has been lost on the authors of the document.

The Nuremberg Code introduced four principles which were not directly covered by the 1931 guidelines. Two of these (No. 4 and 9) are absolutely essential and no fault can be found with them. Articles 5 and 10 have been badly worded and provide a loop hole for investigators, to perform risky trials and continue them when serious harm or death is likely in subjects.

## CONCLUSION

The Nuremberg Code has no legal force behind it, and it would be erroneous even to credit it as the framework on which all future codes have been based. Considering that it was prepared by legal luminaries of that time, it appears to be a poor improvisation over the 1931 Guidelines on human experimentation. It has received far more attention than it ever deserved, probably because it was made in a momentous period and that it was authored by Americans.

The last word in ethics has not been written; even the latest version of the Declaration of Helsinki needs some changes and will undergo numerous revisions with time. Ethics is an ever evolving subject,^[7]^ and repeated revision of ethical codes is evidence of improving human morals and values.
